# Social Inclusion and Communality of Volunteering: A Focus Group Study of Older People’s Experiences

**DOI:** 10.3390/ijerph19095141

**Published:** 2022-04-23

**Authors:** Utta Tiittanen, Riitta Turjamaa

**Affiliations:** 1 Employment Services, 80110 Joensuu, Finland; utta.kolehmainen@meiliboxi.fi; 2 Unit of Continuous Learning, Savonia University of Applied Sciences, 70210 Kuopio, Finland; 3 Department of Nursing Science, University of Eastern Finland, 70210 Kuopio, Finland

**Keywords:** communality, focus group interview, older people, social inclusion, volunteering, qualitative research

## Abstract

The aim of this qualitative study was to describe the factors that motivate older people to volunteer and how volunteering influences their social inclusion and communality. Data were collected using focus group interviews in 2020–2021 from older people aged 65–81 years (*n* = 38) who had experience of volunteering in the community. The data were analyzed using inductive content analysis. Based on the results, the experiences described by older people who participate in volunteering consisted of factors encouraging older people to volunteer and depended on the activation, support, and motivation of volunteer operators. The support provided by the health care services and volunteer action organization for volunteers was a key factor in encouraging older people to participate in volunteering. Moreover, volunteering impacted the communality of volunteers in several ways, influencing social capital. The sense of communality was enhanced by the way in which volunteering was carried out, including collaboration with health care services and associations. In order to strengthen the importance of volunteering in society, there is a need for close collaboration between voluntary organizations and health care service providers to develop volunteering because health care organizations cannot respond to all of the challenges faced by the ageing population.

## 1. Introduction

Globally, life expectancy is increasing, and many older people living in their own homes are healthier than ever [1,2]. The fact that older people have the opportunity to be involved in the community creates a sense of inclusion and reduces loneliness [3,4]. In addition, the networks, empathy, and trust created through social activities have been identified as good ways to prevent the exclusion of older people [5,6]. At the same time, the healthcare sector is attempting to meet the challenging situation resulting from the growing number of older people who need safe healthcare services [7,8]. It is a health policy goal to find new ways to support aging population living at home [9]. One opportunity is to strengthen voluntary work in collaboration with associations [10].

Volunteering has been described as unpaid work done in voluntary organizations to support healthcare services in the municipality to help older people by producing meaningful activities among older people in the community [11]. Volunteering has a wide range of meanings for older people and for society. It encourages and inspires the activity of those involved and intertwines inclusiveness and a sense of communality [12]. Older people describe volunteering as activities that involve learning from others and receiving group support as well as communal support. Human capital, networks, trust, and inclusion also increase [13]. In addition, older people feel that they have the opportunity to make a difference and bring out ideas of their own. Being involved in volunteering brings experiences of communality [14,15].

Based on earlier studies, the experience of communality and inclusion can have numerous effects on the overall well-being of older people, including opportunities to live a socially active life [13,15]. Older people have been reported experiencing communality related to security, familiarity, and amenity, with an emphasis on interactive social support networks. Communities also include respect for common norms and values [16]. In addition, the inclusion of older people has been described through tangible and intangible dimensions. The immediate surroundings of everyday life form the material experience of the deepest inclusion [17], concerning neighbors and age-friendly living environments that are accessible and thus support the inclusion of older people [18]. Moreover, strengthening and reciprocating the inclusion of older people brings relevance to their lives through communal action [19,20]. This involves organizing activities in an accessible vicinity by taking care of assistance and services in everyday life that are also associated with material inclusion [21]. This intangible dimension of inclusion includes social relations, incorporating social skills and the experience of reciprocity in everyday life. These social skills reinforce the experience of inclusion and, in turn, contribute to being part of the community and acting in it. The experiences of inclusion are influenced by the individual’s own resources as well as the structure and atmosphere of society [12]. On the other hand, they can support or hinder the realization and experience of individual inclusion. However, participation has been described as an essential issue in terms of older people’s views of inclusion [15,20].

From a societal point of view, it is significant to understand that, in terms of supporting the inclusion and comprehensive well-being of older people in the society, the volunteering of older people has the same societal importance as the social and healthcare service [13,15]. Research has shown that developing volunteering opportunities that affect volunteerism plays an important role in recognizing older people’s own goals to realize volunteering [4]. Therefore, healthcare professionals working in the municipalities play an important role in recognizing the needs of volunteering, identifying the resources of volunteering older people, and supporting them to achieve significant meaning in their volunteering role [13].

Most recent studies have focused on investigating the effects of volunteering on the well-being of older people [3,15,16]. More knowledge and understanding of the reasons for older people’s volunteering are needed. This will help the health care sector in collaborating with associations to develop ways of volunteering among older people and in finding new models to realize and support older people in their volunteering. Therefore, the aim of this study was to explore the factors that motivate older people to volunteer and how volunteering influences their social inclusion and communality.

## 2. Materials and Methods

### 2.1. Study Design

This was a qualitative focus group study of older people who volunteer. A focus group study was chosen because participants have the opportunity to talk and reflect aloud on their thoughts and answers, generating data about the subject studied. This method is useful to find answers about shared and individual experiences [22]. The study was carried out in the rural region of eastern Finland.

### 2.2. Research Environment, Study Participants, and Data Collection

The study was carried out in in two towns during years 2020–2021. These cities are located in the rural region of eastern Finland. The number of residents in the cities is 75,000–121,557, and the number of older people (65 year or over) is 16,900–119,000 [23]. In Finland, voluntary work among older people is organized, in most cases, in collaboration with local municipalities and the not-for-profit sector, such as associations. Home care services for older people mainly focus on supporting older people in their daily activities. In addition, support services, such as cleaning and meals-on-wheels services, complement home services when needed [24]. Therefore, social activities for older people are organized in collaboration with associations such as the Finnish Association for the Welfare of Older People, the Finnish Red Cross, and the Alzheimer Society of Finland. Voluntary work among older people is common; 58 percent of older people have volunteered during the year [25].

Two researchers presented the study to older people at meetings of volunteers in two cities. Older people were informed both orally and in writing about the aims of the study, conducting focus group interviews, and the voluntary and confidential nature of their participation. A total of 52 older people received the information, and 38 of them expressed willingness to participate in the study. Those that volunteered to take part provided written informed consent. The researchers contacted the older people to schedule an appropriate day for the focus group interviews.

The study participants were 38 retired older people—28 females and 10 males were happy to volunteer to take part. Three of the participants had no previous experience of volunteering, and fourteen had more than ten years’ experience of volunteering. The participants were Finnish by ethnicity, and they lived in two rural towns. They were 65–81 years old, and their average age was 76.8 years; they had been teachers, private entrepreneurs, bank employees, chief executive officers, shop assistants, engineers, nurses, and social workers by profession (Table 1).

The focus group interviews were conducted at the Office of Volunteers in two towns in eastern Finland during the years 2020–2021. Eight focus groups, each with two to six participants (*n* = 38), were conducted by the two researchers. The interviews were conducted and recorded by the two researchers. An interview guide was designed based on previous literature of older people’s volunteering. It consisted of an introduction, in which the researchers recalled the volunteerism of participating in the study; a discussion, which included themes; and a conclusion. At the beginning of the focus groups, the researchers went through the interview guide to establish a confidential relationship with the participants. The researchers also reminded participants of the voluntary nature of the study and that any comments they made would be reported in a way that respected their identity and need for personal confidentiality. They were asked about their background, such as their age, their occupation, and the length of volunteering experience. Three themes were explored during the focus groups (Table 2). The recorded focus groups lasted for 35 to 130 min.

### 2.3. Data Analysis

The focus group interview data were analyzed using inductive content analysis [23,24]. The recorded data were transcribed by two researchers. The data consisted of transcribed text covering 58 pages of A4 paper using 1.5 line spacing. The first step was to read through all the text to understand the content of the interviews. The second step was to modify the transcribed text into units of meaning, namely a single word, combination of words, sentences, or a whole paragraph. After that, the data were grouped into sub-categories (e.g., social relationships), upper categories (e.g., mutual activation), and main categories (e.g., factors encouraging volunteering). The final phase of data analysis was conducted together with the two researchers [26,27].

### 2.4. Ethical Considerations

The ethical principles of the Declaration of Helsinki (2018) were followed throughout the study. The Ethics Committee of the Savonia University of Applied Sciences provided ethical approval, and the participating associations granted the required research permission [28]. Before participating in the study, all participants received verbal and written information about the study and were told that participation was voluntary and that they could withdraw from the study at any time. Participants were told that the interview material was confidential, and only the researchers had access to it. They were also told that the study results would be reported in such a way that their identity and confidentiality would be respected. After being provided with information about the study, the participants were given time to consider whether they wanted to take part. Finally, written informed consent was obtained from all participants. Any data were kept in a password-protected computer.

## 3. Results

Based on the results, the experiences described by older people who participate in volunteering consisted of factors encouraging volunteering and communality and social inclusion as social capital.

### 3.1. Factors Encouraging Volunteering in Older People

The analysis showed that factors encouraging older people to volunteer depended on the activation, support, and motivation of volunteer operators (Table 3). Volunteering activates both the older people who are volunteer operators and the older people who are volunteering, which can be described as mutual activation. Volunteer operators said that they were able to take advantage of earlier work experience before retirement age. For example, a volunteer operator made crafts in her work. She described being able to use these skills for common crafting moments with older people. In addition, as volunteer operators, older people also sang along with those involved in volunteering activities in the clubrooms of the department houses. Some of them described that they were initially just watching in the background, and after two weeks, they sang independently.

*“I’m pretty shy and gladly watch other people do things. However, after a while I found myself in on the singing. Nice group encouraged me like others, too”. (Volunteer 8)*

Older people explained that participating in volunteering activates their social relationships and brings rhythm and meaningful experiences to their life. Outdoor activities are also an expected event for everyone involved in the activities, including caregivers. At the same time, it is possible to go to coffee, where you might meet old friends. This has also activated the emergence of a confidential family relationship, which has arisen between volunteer operators and the people being helped. Voluntary operators also help the caregivers if they need some help with everyday activities. However, they emphasized that they help when they have time.

The support provided by the health care services and volunteer action organization for volunteers was a key factor in encouraging older people to participate in volunteering. One way to support volunteers was through education in collaboration with different volunteering groups from different volunteering fields. Education included issues such as “what volunteering is” and “what it means to become a volunteer”. In addition, there was a discussion on the concealment of confidential information in volunteering. After the training, volunteers were allowed to choose which type of volunteering they wanted to join. In addition to this, a few further hour-long education sessions were carried out for volunteers, such as first aid training and how to handle a person with a memory disorder. These sessions were performed by professionals in the social and health care field, focusing on acting specifically from the point of view of volunteers.

*“We had co-training with the congregation at the parish centre. We went through practicalities and how to approach voluntary activities and what is the theoretical basis”. (Volunteer 1)*

Older people described that they have various ways to become involved in volunteering. One of those interviewed had been chatting with another older person, who was acting as volunteer, at a lunch meeting and was then enthusiastic about taking part in volunteering. Another of those interviewed was inspired by a friend to become involved in volunteering through a personal request. The visibility of volunteering in the local newspaper, with information about what was done in the field of volunteering, also inspired older people to volunteer. Furthermore, the article mentioned that there was a need for new volunteers.

Older people perceived the support of the volunteering coordinator as one of the more significant forms of support. They stressed the importance of always having a support person involved when the volunteer meets the person that they will be helping for the first time. Older people also felt that it is important that the support person is easily available and can be quickly contacted by email or phone. They also actively inform about future joint events via email. Older people felt that engagement was also strongly accompanied by personal feedback from volunteering. They felt that they often received personal feedback that helped them develop as volunteers.

*“When I compare this to working life, there was almost never feedback there. But this is where you get all the time. And it probably encourages more all the time. And have mental support, I mean project person”. (Volunteer 18)*

All volunteers said that participation in volunteering can be divided into motivational factors from themselves and from the outside. These factors together constitute a motivational entity for volunteering. Reasons from the volunteers themselves inspire them to carry out and continue the volunteering that has already begun. The most significant reason to volunteer is to have a good attitude, and this comes from helping other older people. Seeing the visible pleasure in the person you are helping is also significant.

Emotional motives encouraged volunteers. They explained that another person needing help creates a desire to help. Experiencing a sense of need was described as grounds to volunteer, and life values played a big part. Interviewees described material needs as being sufficient; however, this feeling was immaterial but gave them a great deal. In addition, reflection on their own future prompted them to act as volunteers. One of those interviewed wondered that she was the youngest of the siblings: some of the older siblings are already in need of help; in the future, she may need help with everyday activities.

*“Becomes a good mind when you get someone to go out and see that a good mind comes to them too. We both benefit from this”. (Volunteer 32)*

Older people emphasized the importance of life values as one factor motivating volunteering, such as the desire to help. Some of the volunteers thought about their own life values and recognized that they have had an impact through having participated in volunteering. Other factors encouraging volunteering included a desire to learn something new, challenging oneself, and reminiscing. In addition, older volunteers reported that volunteering was interesting and brought a counterweight to their own life situation. Many of the older people stressed that they have more time in retirement to do things that interest them. One of those interviewed had just retired and longed to still participate in life. His wife was still working, so he had plenty of time. Volunteers also said that they received a great deal of positive feedback about their work, which was perceived to be one key motivating factor. Older volunteers appreciated the togetherness between themselves and those who were helped, which allowed confidential conversations and humor to be shared. Social relationships with other older people was the most significant factor motivating volunteering. Some of the volunteers lived alone, and the action brought them new friends. Conversations, joint meetings, peer support, and an experience of parity with other older people encouraged action.

### 3.2. Communality and Inclusion as Social Capital

Based on our results, volunteering impacted the communality of volunteers in several ways, influencing social capital (Table 4). The older people felt a sense of community, which was strengthened by common rules that everyone respected. Joint meetings for volunteers were particularly significant, where they were free to speak about their experiences in volunteering. The sense of communality was enhanced by the way in which volunteering was carried out. Some of those interviewed wanted to volunteer as a group and some on their own: some felt that doing something in a group made it easier because they received support from other volunteers, while others felt that doing it alone and in their own way was the best way to participate in volunteering. In the latter case, they felt that they could independently decide on things that were perceived to be relevant in volunteering.

*“By supporting each other, and then we get yourself. After all, we have visited the theatre and we have been eating together”. (Volunteer 13).*

The sense of communality was also reinforced by joint action excursions, and shared lunches were held for volunteer operators, where all volunteers were welcomed. Joint meetings create a good sense of togetherness among volunteers. On the other hand, volunteers considered that the sense of community has diminished from society as a whole. Interviewees described the sense of community as having changed, and even disappeared, from society.

Volunteering strengthened the inclusion among volunteers, but some negative past experiences of volunteering have undermined inclusion. All of these experiences have an impact on social capital. Volunteers reported that social interactions with others fostered inclusion. The experience of inclusion came to fruition in encounters with volunteers and recipients of the support. Some volunteers were living alone, so being involved in volunteer activities increased social contact. Couples also reported that new social contacts had a positive effect on their mood even though they did not experience loneliness like the volunteers who were living alone.

The positive change in attitude that took place by being involved in volunteering also reinforced the experience of inclusion. Interviewees described having gained a different perspective on life through voluntary activities. It is important every day to live through positivity. The sense of inclusion was also reinforced by new experiences that saw volunteers gaining insight on their own lives, such as relationships. Inclusion was also strengthened by the use of their own knowledge and skills in the voluntary activities. The preservation of professionalism and its use in voluntary activities was perceived as necessary and important.

*“I enjoy meetings with my friends. We can discuss almost everything. I can help with electrical work, thereby maintaining professionalism”. (Volunteer 5)*

Conversely, volunteers reported that issues that negatively affect inclusion have also been raised when volunteering. For example, an article in the journal gave a negative picture of volunteering, and the volunteers developed a feeling that the work done by the volunteers was not appreciated. However, volunteers stated that they have encouraged their friends to join them in volunteering because there is a great need for it in society.

## 4. Discussion

In this discussion, we focus on volunteering as a promoter of communality and social inclusion because our results indicated that voluntary activities had a wide range of implications for communality and the social inclusion of older people.

The older volunteers said that volunteering filled the social void. Our results are similar to those indicated by previous studies where experience of participating in volunteering has been found to affect the overall well-being of older people, including opportunities to live a socially active life [13,15]. Volunteering has been recognized to support older people in many ways, which has reinforced a sense of communality and social inclusion, but there have also been factors that have undermined their experience. It is evident that older volunteers are the best people to say what effects their sense of communality and social inclusion. If older volunteers feel they are going to be heard, this has an impact on social capital and, by extension, on well-being.

According to our results, volunteers felt welcome in the group of volunteers, and this reinforced the sense of communality among older people. A good sense of togetherness and the support of the volunteers created a sense that they were accepted and respected members of the community. In volunteering, both volunteers and those who are helped experience feelings of reciprocity, empowerment, and inclusion. This can be described as an increase in the welfare and social capital of volunteers [15]. Communality in the form of voluntary activity also strengthened the growth of social capital and became an asset for the individual and the community, increasing the well-being of both. As the community grew, the meaning of volunteering changed. It began as traditional outdoor activities but later changed to a shared, confidential family relationship. In previous studies, some volunteer actors began volunteering in order to remain active, but the motive turned into an altruistic one [18]. On the other hand, some older people wanted to keep the aided volunteer operator deliberately remote in the relationship so that they did not have to be thought about at any other time. Interviewees wanted clear limits on when they participated in volunteering.

The experience of inclusion came to fruition in encounters between volunteers and the recipient of the support. Although some of the people who were helped were unable to speak or otherwise participate in joint activities, for example, older people with a memory disorder, volunteering made it possible for them to go out and have coffee and encounter acquaintances there. This requires that volunteers show consideration and can reconcile the views of the expert and those who were helped in such a way that the best interests are met. In the encounter between volunteers and family caregivers, the idea that there are opportunities for participation is essential for inclusion. In addition, volunteers were able to influence the well-being and health of the local community by their own actions. This contributed to the solidification of the volunteers’ sense of inclusion. Previous research has found inclusion to be strongly linked to communities, the environment, and society. The experiential elements of everyday inclusion arose from a wide range of issues, including being heard, as well as the encounters, social relationships, and influence on things in one’s life and making decisions about them. Furthermore, inclusion strengthened commitment to the community and its activities through voluntary activity [12].

In this study, participation in volunteering did not arise from the perspective of self-fulfillment or from the need to maintain one’s role in the community. In contrast, long-term volunteering has been linked to maintaining a role in the community and has also been identified in terms of solidarity. A volunteer helps another human being to promote a common interest. Neither was the interviewees’ motive to participate in volunteering related to the physical factor associated with residential areas, as has been described in previous study [13].

Previous studies showed that various motives are influenced by volunteering, such as the desire to engage in meaningful work [20,29] or financial and material incentives [15,30]. However, our study participants were older people. They said that values, sociability, and their own development encouraged them to join volunteering, but safety and career did not come up as motivations. This may be because of the age of our volunteers and because volunteering in Finland is not done for the purpose of benefiting the career; Finland is also considered to be a safe country to live in.

The results showed elements that promote older volunteers’ social inclusion and communality based on their individual skills and abilities as highlighted by voluntary work. One essential question concerns the factors that contribute to older peoples’ volunteering. As populations are ageing, it is evident that municipal services for older people who need help with everyday activities and social relationships cannot respond to the challenges without collaboration with non-profit sector, such as voluntary work.

## 5. Study Limitations

There are some limitations to our study that warrant attention. Our study was carried out among a small group of Finnish, community-dwelling older volunteers; therefore, the results cannot be generalized without further studies that are focused on the role of volunteering as a promoter of communality and inclusion among older volunteers. The method used involved focus group interviews. It is possible that individual interviews would have provided a deeper insight into the topic. However, we used focus groups as a data collection method because this approach is successful in achieving diverse perspectives and multidimensional views about the experiences of older volunteers. Thus, the large number of opinions of the participants gives confidence in the results. In pondering the benefits of this study, it was agreed that the results could possibly trigger discussion among health and social sectors and volunteer operators to support inclusion and the comprehensive well-being of older people in the community.

## 6. Conclusions

The results of our study provide information regarding the experience of older volunteers with respect to the factors that motivate them to volunteer and how volunteering influences their social inclusion and communality. Our results highlight several elements indicating that voluntary activities have a wide range of implications for communality and social inclusion of older people, such as activation, support, and motivation of volunteer operators. In addition, volunteering impacted the communality of volunteers in several ways that influenced social capital. The older people felt a sense of community, which was strengthened with common rules that everyone respected. However, the volunteers’ perspective was limited to collaboration with volunteer operators and the people who were helped. In order to strengthen the importance of volunteering in society, older volunteers need more support from the municipality. In addition, there is a need for close collaboration between voluntary organizations and community service providers to develop volunteering because community service organizations cannot respond to all the challenges faced by the ageing population.

## Figures and Tables

**Table 1:** Demographics of the study participants.

Demographics of Participants		*n* = 38	
Gender			
Female		28	
Male		10	
	Mean	Min	Max
Age	76.8	65	81
Occupation field		*n*	
Social and health care, Education, Finance and marketing, Technology		18, 11, 6, 3	
Length of volunteering	0 3	1–9 year 21	<10 years 14

**Table 2:** An interview guide for the focus groups.

Themes
(1) What motivates to join in volunteering? (2) How has volunteering affected a volunteer’s sense of communality? (3) How has volunteering affected a volunteer’s inclusion?

**Table 3:** Factors Encouraging Volunteering in Older People.

Main Categories	Upper Categories	Sub-Categories	Examples of Simplified Sentences by the Study Participants
Factors Encouraging Volunteering	Mutual activation	Social relationships (volunteer 34/38) Meaningful experiences (volunteer 37/38)	“I made new friends and meaning for life.” “I’m glad I can help other people with my own activities.”
	Support	Encouragement to participate in volunteering (volunteer 29/38) The significance of the volunteering coordinator (volunteer 38/38)	“Some of my friends encouraged me to become involved in the voluntary work.” “Especially at the beginning of volunteering, support of the volunteering coordinator encouraged me to continue.”
	Motivation	Good feeling about helping others (volunteer 37/38) Importance of life values (volunteer 36/38)	“I’ll be happy when another person becomes happy with me.” “After retiring, I needed content and meaning for life.”

**Table 4:** Communality and social inclusion as social capital.

Main Categories	Upper Categories	Sub-Categories	Examples of Simplified Sentences by the Study Participants
Communality and social inclusion as social capital	Increased sense of community	Togetherness and support (volunteer 33/38) Joint regular meetings (volunteer 36/38)	“By supporting each other, and then we get yourself.” “After all, we have visited the theatre and we have been eating together.”
	Strengthening inclusion	Sociability (volunteer 36/38) Preservation of professionalism (volunteer 21/38)	“I enjoy meetings with my friends. We can discuss almost everything.” “I can help with electrical work, thereby maintaining professionalism.”

## Data Availability

All data generated or analyzed during this study are included in this manuscript.
